# C77G in *PTPRC* (CD45) is no risk allele for ovarian cancer, but associated with less aggressive disease

**DOI:** 10.1371/journal.pone.0182030

**Published:** 2017-07-31

**Authors:** Johannes Landskron, Sigrid M. Kraggerud, Elisabeth Wik, Anne Dørum, Merete Bjørnslett, Espen Melum, Øystein Helland, Line Bjørge, Ragnhild A. Lothe, Helga B. Salvesen, Kjetil Taskén

**Affiliations:** 1 Centre for Molecular Medicine Norway, Nordic EMBL Partnership, University of Oslo and Oslo University Hospital, Oslo, Norway; 2 K.G. Jebsen Centre for Cancer Immunotherapy, University of Oslo, Oslo, Norway; 3 Department of Molecular Oncology, Institute for Cancer Research, Oslo University Hospital, The Norwegian Radium Hospital, Oslo, Norway; 4 Center for Cancer Biomedicine, Faculty of Medicine, University of Oslo, Oslo, Norway; 5 K.G. Jebsen Colorectal Cancer Research Centre, Oslo University Hospital, Oslo, Norway; 6 Department of Pathology, Haukeland University Hospital, Bergen, Norway; 7 Centre for Cancer Biomarkers CCBIO, Department of Clinical Medicine, Section for Pathology, University of Bergen, Bergen, Norway; 8 Department of Gynaecologic Oncology, Oslo University Hospital, The Norwegian Radium Hospital, Oslo, Norway; 9 Norwegian PSC Research Centre, Department of Transplantation Medicine, Division of Surgery, Inflammatory Medicine and Transplantation, Oslo University Hospital, The National Hospital, Oslo, Norway; 10 K.G. Jebsen Inflammation Research Centre, University of Oslo, Oslo, Norway; 11 Department of Obstetrics and Gynaecology, Haukeland University Hospital, Bergen, Norway; 12 Department of Clinical Science, Centre for Cancer Biomarkers, University of Bergen, Bergen, Norway; University of South Alabama Mitchell Cancer Institute, UNITED STATES

## Abstract

The pan lymphocyte marker CD45 exists in various isoforms arising from alternative splicing of the exons 4, 5 and 6. While naïve T cells express CD45RA translated from an mRNA containing exon 4, exons 4–6 are spliced out to encode the shorter CD45R0 in antigen-experienced effector/memory T cells. The SNP C77G (rs17612648) is located in exon 4 and blocks the exon’s differential splicing from the pre-mRNA, enforcing expression of CD45RA. Several studies have linked C77G to autoimmune diseases but lack of validation in other cohorts has left its role elusive. An incidental finding in an ovarian cancer patient cohort from West Norway (Bergen region, n = 312), suggested that the frequency of C77G was higher among ovarian cancer patients than in healthy Norwegians (n = 1,357) (3.0% vs. 1.8% allele frequency). However, this finding could not be validated in a larger patient cohort from South-East Norway (Oslo region, n = 1,198) with 1.2% allele frequency. Hence, C77G is not associated with ovarian cancer in the Norwegian population. However, its frequency was increased in patients with FIGO stage II, endometrioid histology or an age at diagnosis of 60 years or older indicating a possible association with a less aggressive cancer type.

## Introduction

CD45 (PTPRC, leukocyte common antigen) is a receptor-like protein tyrosine phosphatase expressed on the surface of all leukocytes. Several isoforms of the glycoprotein exist, arising from alternative splicing of the exons 4, 5 and 6 (also named A, B and C) in the CD45 pre-mRNA. In the T cell compartment, naïve peripheral T cells express heavy isoforms usually containing exon 4 (A) (CD45RA), optionally in different combinations with the exons 5 and 6. Upon stimulation, the expression profile changes and activated as well as memory T cells exclusively express the light isoform, CD45R0, without exons 4–6 [1, 2]. The main substrates of the CD45 protein phosphatase are the Src family kinases (Lck in T cells) that become activated upon CD45-mediated dephosphorylation of their C-terminal tyrosine [2] and the Janus kinases JAK1 and JAK2 [3] activating members of the Stat family of transcription factors such as Stat5, making CD45 an important regulator of T cell signalling and activation.

Already 20 years ago the base exchange C77G (rs17612648) in the *PTPRC* gene was described as the SNP responsible for the lack of CD45R0^+^ (CD45RA^-^) T cells in a small proportion of blood donors [4–6]. While not altering the protein sequence, C77G mutates an activation-responsive sequence (ARS) within the exonic splicing silencer 1 (ESS1) of exon 4, which normally enables expression of CD45R0, thereby enforcing the expression of CD45RA [7, 8]. As a functional consequence, C77G bearing CD4^+^ T cells are hyperactive compared to their counterparts expressing only the major allele. While expressing similar amounts of Lck, the inhibitory phosphorylation of Lck at Y505, a target for CD45 phosphatase activity, is reduced in C77G carriers resulting in a larger pool of active Lck. This leads to increases in phosphorylation of CD3ζ and ZAP70, Ca^2+^ flux as well as augmented production of the cytokines IL-2, IL-4 and IL-10 [9]. Furthermore, C77G cells proliferate more in response to TCR/CD28 (only the memory CD4^+^CD45R0^+^ cells) and IL-2 stimulation [9, 10]. However, an opposite, hypo reactive effect of C77G was recently described for regulatory T cells [11].

Following its identification, the C77G SNP has been linked to various autoimmune diseases like multiple sclerosis (MS) [12, 13], autoimmune hepatitis [14] and systemic sclerosis [15]. However, since several follow-up studies failed to confirm correlations and it has not been reported in any genome wide association study (GWAS) [16–22], the role of C77G in autoimmune diseases remains elusive [23].

Like in autoimmune diseases, genetic predisposition plays an important role in the development of cancer. In the case of ovarian cancer, which is the 7^th^ most common cancer in women world-wide with an estimated age-standardised rate (ASR) of 6.3 per 100 000 [24], it is known that germ line mutations in *BRCA1* and *BRCA2* increase the cancer risk in affected families dramatically. Recent GWASs in ovarian cancer discovered several new loci, where the presence of the SNPs increases cancer susceptibility [25–30]. In the present study, we evaluated a potential association of C77G in *PTPRC* with ovarian cancer in Norwegian patients.

## Materials and methods

### Patient samples and healthy controls

Ovarian cancer patient samples from West Norway (Bergen region) consisted of an initial patient cohort (n = 17), where blood and ascites samples were collected in parallel [31] and a biobank of 312 patient samples, blood-derived/germline DNA collected between 1993 and 2009 [32]. DNA from 15 patients of the initial cohort was also present in the DNA biobank. Both cohorts were collected at the Haukeland University Hospital in Bergen, Norway. The validation cohort was blood-derived/germline DNA from 1,198 ovarian cancer patients from South-East Norway (Oslo region). They were collected between 2002 and 2012 at The Norwegian Radium Hospital, Oslo University Hospital, Norway [32]. Clinicopathologic parameters were available for all patients. Data from 1,357 healthy Norwegian controls was provided by the International PSC Study Group [33]. These samples had already been genotyped using the Illumina_Immunochip (Illumina), which contains C77G. The study protocol has been approved by the regional ethics committees for south-east Norway (Regionale Komiteer for Medisinisk og Helsefaglig Forskningsetikk, REK sør-øst), approvals REK 2014/473, NSD15501 and REK 052.01, and west Norway (REK vest) approval REK VEST 2009/2315. Samples were collected after written informed consents were signed.

### Flow cytometry

Cells from the patient ascites fluid were isolated as described previously [31]. In brief: after filtration of the ascites fluid through a 40 μM mesh, cells were sedimented by 10 min centrifugation at 350 g. Then, cells were fixed and permeabilised using the Human FoxP3 Buffer Set (BD), stained with CD3-Pacific Blue (UCHT1, BD Biosciences, AB_397038), CD4-PerCP (L200, BD Biosciences, AB_393791), CD8-PE-Cy7 (RPA-T8, BD Biosciences, AB_396856) and CD45RA-APC-H7 (HI100, BD Biosciences, AB_1727497) and run on a FACSCanto II (BD Biosciences). Data visualization and analysis was done in FlowJo (V10) (FlowJo, LLC).

### RFLP and sequence analysis

RFLP (restriction fragment length polymorphism) analysis was performed as described previously [34] utilizing a novel *Msp*I restriction site [4]. Briefly, a 155-bp genomic DNA region containing the SNP was amplified by PCR using forward (GACTACAGCAAAGATGCCCAGTG) and reverse (GGGATACTTGGGTGGAAGTA) primers in an AccuPrime *Pfx* SuperMix (Thermo Fisher Scientific) reaction with 63°C annealing temperature. After digestion with *Msp*I the fragments were separated on a 10% polyacrylamide gel (Criterion TBE, Bio-Rad) or sent for sequencing using the same primers.

### KASP genotyping assay

KASP (competitive allele specific PCR) is a genotyping technology based on allele specific oligo extension that uses fluorescence for signal generation. It was performed by a genotyping service at the Department of Neurology at the Oslo University Hospital on a ViiA7 platform (Thermo Fisher Scientific) using a LGC-designed (LGC, Teddington, UK) assay running a 10 cycle touchdown protocol (94°C for 20 s and 61–55°C for 60 s with 0.6°C/cycle) followed by 26 cycles with 94°C for 20 s and 55°C for 60 s. RFLP-genotyped patient samples from the Bergen cohort were used a C77G heterozygous and homozygous controls.

### Statistical analysis

Categories were compared using Pearson’s *χ*^2^-tests and Fisher’s exact tests where appropriate. Odds ratios with 95% confidence intervals were estimated. Mann-Whitney U and *t*-tests were applied when comparing distribution of continuous variables between groups. Univariate disease-specific or overall survival analyses of time to death were performed using the Kaplan-Meier method. Entry date was the time of primary surgery. For the Bergen cohort, patients who died from other causes were censored at the date of death. For the Oslo cohort, only overall survival data was available. Differences in survival between groups were estimated by two sided log-rank (Mantel Cox) tests. GraphPad Prism 6 (GraphPad Software, La Jolla, CA, USA) was applied for the statistical analyses. Statistical significance was assumed for P values < 0.05.

## Results

**Fig 1:**
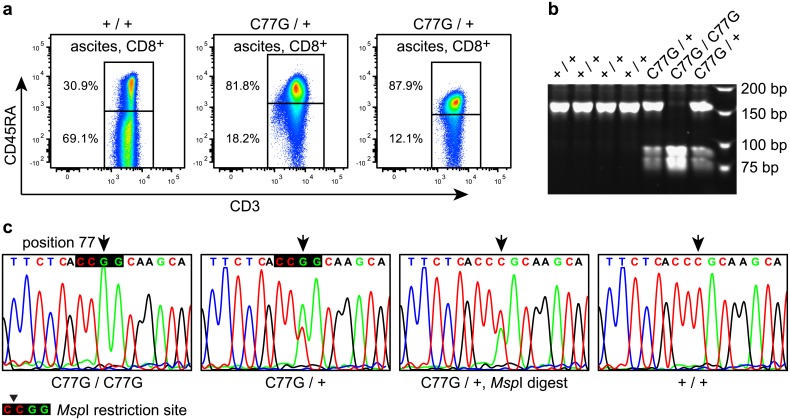
Flow cytometry of patient samples, RFLP and sequence analysis. (a) Flow cytometry analysis of ascites fluid derived CD3^+^CD8^+^ TALs from one SNP-negative (+/+) and two C77G-heterozygous (C77G/+) ovarian cancer patients, the latter showing reduced numbers of CD45RA^-^ cells in ascites fluid. (b) RFLP analysis of four SNP-negative (+/+), two C77G-heterozygous (C77G/+) and the C77G-homozygous (C77G/C77G) patient. Upper bands represent the undigested 155-bp PCR fragment whereas the two lower bands are the *Msp*I restriction fragments of 83 and 72 bp, when C77G is present. (c) Sequence analysis of PCR products of the homozygous (C77G/C77G), one heterozygous (C77G/+) before and after *Msp*I digest and one SNP-negative (+/+) patient. Note that sequencing of the heterozygous patient results in a double peak C/G at position 77 favoring G, while after *Msp*I digest the double peak is reversed.

During a study in 17 ovarian carcinoma patients from West Norway (Bergen region), comparing different CD3^+^CD4^+^ and CD3^+^CD8^+^ tumour-associated lymphocyte (TAL) populations derived from the ascites fluid with patient peripheral blood mononuclear cells (PBMCs) [31], two patients were incidentally discovered to display an unusual distribution between naïve and memory T cells. While all other patients showed a shift towards the CD45RA^-^ memory compartment, indicating increased levels of antigen-experienced TALs, these two patients seemed to have dramatically reduced numbers of memory T cells in blood and more surprisingly in the ascites fluid (Fig 1a, middle and right panels). The majority of CD3^+^CD4^+^ and CD3^+^CD8^+^ T cells in these two patients was positive for CD45RA (CD3^+^CD8^+^: 82% and 88%, compared to 31% in an average patient) (Fig 1a, left panel) expressing a CD45 splice variant containing exon 4 (exon A). We therefore hypothesised that the observed pattern could be related to the SNP C77G (rs17612648) in the *PTPRC* gene, which is known to enforce the expression of CD45RA. Hence, we examined germline DNA from the two patients for the presence of C77G with RFLP analysis, utilizing a novel *Msp*I restriction site created by the C to G exchange. This revealed that both patients were heterozygous carriers of the C77G SNP resulting in 5.9% minor allele frequency (MAF) among the initial 17 patients analysed.

**Fig 2:**
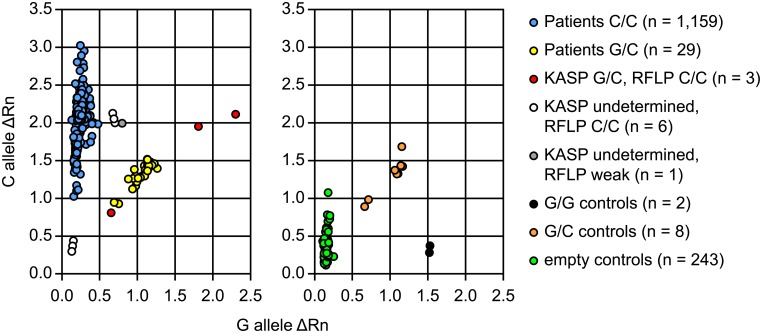
Allelic discrimination plots. Results of the KASP assay for the Oslo cohort (n = 1,198 ovarian cancer patients) from South-East Norway (left plot) and controls (right plot). RFLP-genotyped patient samples from the Bergen cohort were used as SNP heterozygous and homozygous controls. All samples evaluated by KASP as heterozygous or undetermined were re-analysed by RFLP. Patients C/C: SNP negative, Patients G/C: heterozygous C77G/+, KASP G/C, RFLP C/C: scored as heterozygous with KASP and SNP-negative with RFLP, KASP undetermined, RFLP C/C: scored undetermined with KASP and SNP-negative with RFLP, KASP undetermined, RFLP weak: scored undetermined with KASP and showed only a weak signal with RFLP (added to the SNP-negative group). ΔRn: difference of the normalised fluorescent reporter signals in the KASP assay.

**Fig 3:**
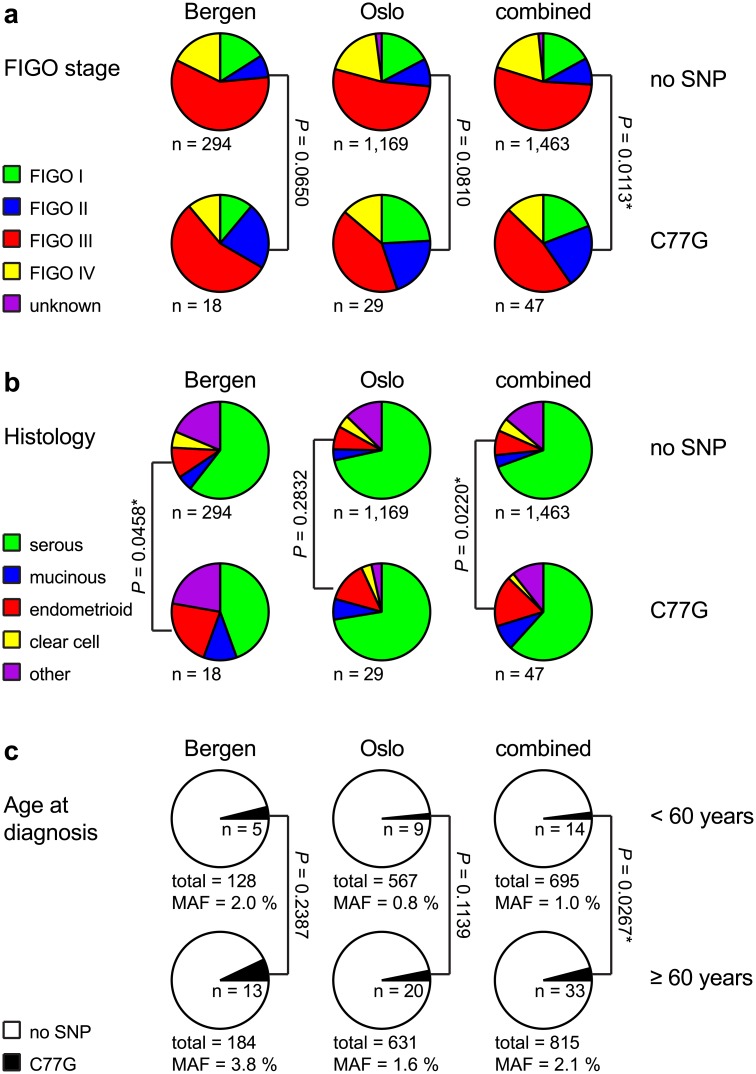
Clinicopathologic parameters of the cohorts. Distribution of FIGO stages (a) and histologic subtypes (b) within the Bergen, Oslo and combined cohorts. Pie charts display both the respective C77G-negative and the C77G-positive patients. (c) Number of SNP carriers within the patient subpopulations with an age at diagnosis < 60 and ≥ 60 years. All P values (*χ*^2^) are based on allele frequencies. Significant P values are indicated by asterisks. MAF: minor allele frequency.

For further evaluation, a germline DNA biobank of 312 primary ovarian carcinoma patients collected at the same university hospital was genotyped by RFLP. This biobank included the two C77G heterozygous and 13 SNP-negative patients of the initial cohort. Seventeen of these patients, including the two previously identified, were genotyped as heterozygous (C77G/+) and one patient as SNP-homozygous (C77G/C77G) (Fig 1b and 1c) resulting in an allele frequency of 3.0%. The abundance of C77G was higher among the ovarian cancer patients compared to healthy controls collected in Norway [33] (MAF: G = 1.8%), but did not reach statistical significance with P = 0.0586, an odds ratio (OR) of 1.7 and 95% confidence interval (CI) 1.0–3.0 (Table 1). For validation, a DNA biobank containing samples from 1,198 ovarian cancer patients treated at the Oslo University Hospital (Radium Hospital site), South-East Norway, was analysed using a KASP genotyping assay. Samples from 1,159 patients were genotyped as homozygous SNP-negative, 32 as C77G heterozygous and seven samples remained undetermined. All SNP-positive, the undetermined and 41 random SNP-negative samples were reanalysed with RFLP. All SNP-negative samples were confirmed. Three of the 32 previously heterozygous samples (C77G/+) turned out homozygous SNP-negative in the RFLP, two outliers with the highest G-allele ΔRn and the sample with the lowest ΔRn values in the heterozygous group (Fig 2). All seven KASP-undetermined samples could be added to the homozygous group, including one showing very weak signals in the RFLP, leading to 1,169 SNP-negative patients and 29 C77G heterozygous (Fig 2). The resulting Oslo cohort MAF of 1.2% was, in contrast to the samples collected in the Bergen region, a bit lower than in healthy controls. In fact, the difference in MAF between the two patient groups was greater (P = 0.0008), than the respective differences to the healthy controls. However, the distribution of clinical parameters like FIGO stage, histology and tumour differentiation were similar between the Bergen and Oslo cohorts. The median age at diagnosis was 62 years in Bergen and 60.6 in Oslo (Fig 3a and 3b, S1 Fig). When allele frequencies of the two patient cohorts were combined, the overall allele frequency of 1.6% was similar to that of the Norwegian controls (1.8%, P = 0.67) (Table 1).

**Table 1:** Allele frequencies of C77G in different cohorts.

Cohort	Bergen cohort	Oslo cohort	Combined cohorts	Norwegian controls
**number of individuals**	312	1,198	1,510	1,357
**number of C77G**	17 CG	29 CG	46 CG	48 G-alleles
**carriers / alleles**	1 GG		1 GG	
**MAF**	3.0%	1.2%	1.6%	1.8%
**OR (95% CI)**	1.7 (1.0–3.0)	0.7 (0.4–1.1)	0.9 (0.6–1.3)	
**P (*χ***^**2**^**)**	0.0586	0.1286	0.6708	

Allele frequencies two independent ovarian cancer patient cohorts (Bergen and Oslo) and a healthy Norwegian control cohort. P values (*χ*^2^) were calculated between the respective ovarian cancer patient cohorts and healthy Norwegian controls. Information about homo-/heterozygosity was not available for the Norwegian controls. MAF: minor allele frequency, OR: odds ratio, CI: confidence interval.

**Fig 4:**
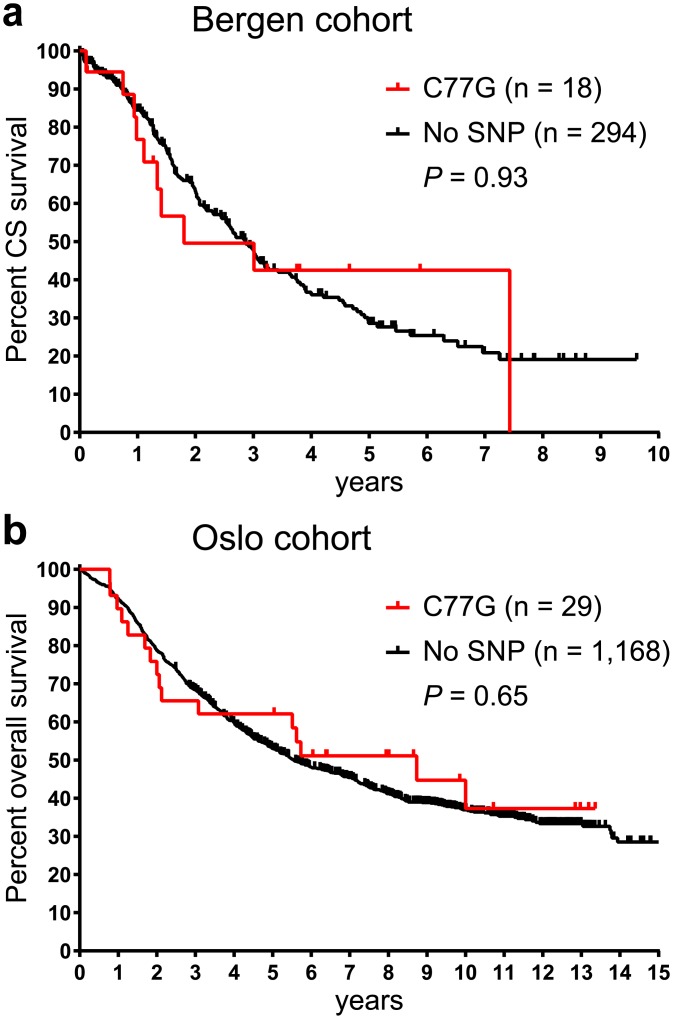
C77G is not associated with patient outcome. Kaplan-Meier plots of Cancer specific (CS) and overall survival curves for the Bergen (a) and the Oslo (b) cohort, respectively. Survival estimates were calculated using two sided log-rank tests (Mantel-Cox). Censored patients are indicated by ticks.

Furthermore, potential associations between clinicopathologic parameters and C77G were explored (summarised in S1 Table). In the combined cohort, the C77G allele frequency in patients diagnosed at FIGO stage II was increased 2.6-fold to 3.6% (P = 0.0113) (Fig 3a) compared to the remaining patients where the allele frequency was 1.4%. Similarly, the C77G allele frequency was increased 2.5-fold to 3.5% in patients with endometrioid tumours (P = 0.0220) (Fig 3b) compared to 1.4% in patients with a different histology. However, there were only two SNP carriers with FIGO stage II that had an endometrioid histology. Both, FIGO stage II and endometrioid histology are independent factors of a relatively good prognosis (S1 Fig). Tumours with an endometrioid histology spread slower compared to the more frequent high grade serous tumours and 69% of these patients were diagnosed at an early FIGO stage I or II compared to only 15% of patients with a serous histology (S2 Fig). Furthermore, patients diagnosed at an age of 60 years or older exhibited a 2.1-fold higher C77G frequency (2.1%) compared to younger patients (1.0%, P = 0.0267) (Fig 3c, S1 Fig). As a consequence, the median age at diagnosis was approx. four years higher in patients carrying the SNP than in non-carriers (S1 Table, S1 Fig). However, the presence of C77G did not influence patient survival in the individual cohorts (Fig 4) and frequencies of C77G did not associate with tumour differentiation (S1 Fig).

## Discussion

In this study, an increased prevalence of the SNP C77G (rs17612648) was initially found in a small ovarian cancer patient cohort from West Norway (Bergen region). This increase did not reach statistical significance in a larger group from the same region and could not be verified in a large Norwegian cohort collected in the south-east (Oslo region). However, finding two heterozygous SNP carriers in a cohort of only 17 patients and an allele frequency of 3.0% in a larger cohort from the same geographic region was very unlikely considering the generally low abundance of this SNP in most healthy populations, ranging from 0–2% with an average of approx. 1% (reviewed in [23]). Therefore it was tempting to hypothesise that C77G could be associated with ovarian cancer risk in general, although the harbouring gene *PTPRC* is not expressed in tumour cells, but restricted to lymphocytes. However, it has been reported earlier that enforced expression of CD45RA by C77G can change T cell function in various ways [9–11], which might also influence anti-tumour immune responses. Furthermore, this was supported by a recent observation that the newly described SNP (rs869736) in the promoter region of *PTPRCAP*, encoding CD45 associated protein (CD45-AP), has been linked to diffuse-type gastric cancer [35]. The base exchange C-309A renders the promoter more active which might lead to higher transcription rate of *PTPRCAP* as well as increased protein levels and activity. Being a positive regulator of CD45 kinase activity [36, 37], this might cause a similar, hyperactive, phenotype like C77G in CD45.

The analysis of a large group of 1,198 ovarian cancer patients from the Oslo region, however, failed to validate the initial observation in the Bergen cohort. The allele frequency in the validation cohort was, with 1.2%, significantly lower than in the Bergen cohort (3.0%) and also lower than in healthy Norwegian controls (1.6%) (Table 1).

Since its discovery twenty years ago, possible disease associations of C77G with predominantly autoimmune diseases like MS, autoimmune hepatitis and systemic sclerosis have been discussed in the literature. The main reason for controversy is the generally low abundance of C77G making association studies difficult. Several initially positive correlations in, by today’s standard, relatively small cohorts (40–300 individuals) could not be reproduced in other studies [23]. C77G has not been reported in any GWAS yet, although it is included in the Illumina_ImmunoChip and Illumina_HumanOmni5 chip. However, GWASs mainly focus on variations with allele frequencies >5%. Furthermore, whereas the allele frequency is low or equals out in larger populations, higher variations are observed in smaller cohorts. Two relatively isolated populations have been reported with higher allele frequencies, but only in comparably small cohorts, Orkney Islands with 3.5% MAF among 72 individuals [38] and the Pamiri people with 6.7% MAF testing 75 individuals [39]. Altogether, the high allele frequencies of 5.9% and 3.0% found in the Bergen cohorts might be incidental findings, but it cannot be excluded that this could also reflect a higher prevalence of C77G in the western part of Norway.

In the present study, C77G was neither associated with the incidence of ovarian cancer in general, nor with patient survival. However, allele frequencies of C77G seemed to be increased in patients diagnosed at the age of 60 or older, in patients diagnosed at a relatively early stage, FIGO stage II, and with cancers of an endometrioid histology. These three observations rather suggest a weak beneficial effect for C77G carriers with ovarian cancer. It might indicate a later onset of the disease and endometrioid histology and FIGO stage II are characteristics of the disease associated with a relatively good prognosis in general. Usually, most patients are diagnosed at advanced disease stages, mainly FIGO stage III due to few uncharacteristic symptoms in the early stages [40]. An augmentation of patients in FIGO stage II might therefore indicate an earlier onset of symptoms in SNP carriers. The endometrioid type represents only ~9% of ovarian cancers and is often confined to the ovaries, while the serous type represents ~70% and most often develop from the tubes and thus spread directly to the peritoneal cavity. It will be interesting to further evaluate a potential beneficial effect of C77G and also to assess whether C77G is of clinical relevance for ovarian cancer patients carrying this variant e.g. with respect to different treatment options. However, even larger datasets will be necessary to address these issues. Nevertheless, this study underlines the necessity of independent validation of potential cancer risk alleles in large patient series of the same ethnicity to claim that a certain SNP conveys a higher cancer risk across different regions presumed to be genetically similar.

## Supporting information

S1 Fig Tumour differentiation and age at diagnosis. Distribution of tumour differentiation (a) and age at diagnosis (c) within the Bergen, Oslo and combined cohorts are displayed both for the respective total number of individuals (upper panels) and the C77G carriers (lower panels). Both, tumour differentiations and age at diagnosis, show similar distributions between the various cohorts. Light grey bars in (c): patients below the age 60, dark grey bars in (c): patient with an age above 60 years. (b) Median age at diagnosis of the Bergen (B., blue boxplots), Oslo (O., red boxplots) and combined (comb., white boxplots) cohorts is approx. four years higher in C77G-positive (striped plots) compared to C77G-negative patients. Boxes range from the first to the third quartiles. The medians are indicated by horizontal lines. Age minimums and maximums are displayed as whiskers. Combined cohorts “no SNP” vs. “C77G”: P = 0.0586.
(PDF)

S2 Fig Survival and FIGO stage distribution within histologic subtypes. Overall survival curves for the different FIGO stages (a) and histological subgroups (b) within the Oslo cohort. Patients with FIGO stages I and II, and endometrioid or clear cell histologic subtypes have a relatively favourable prognosis. (c) Distribution of the FIGO stages within patient groups having a serous, mucinous, endometrioid, clear cell or different (“other”) histologic subtype for the Bergen, the Oslo and the combined cohorts.
(PDF)

S1 Table Clinicopathologic parameters of the cohorts. (PDF)
