# Supplementary figures and images for: C77G in PTPRC (CD45) is no risk allele for ovarian cancer, but associated with less aggressive disease
Source: PLoS One. 2017 Jul 31;12(7):e0182030. doi: 10.1371/journal.pone.0182030 (PMC5536273; doi:10.1371/journal.pone.0182030)

Supplementary Figure S1

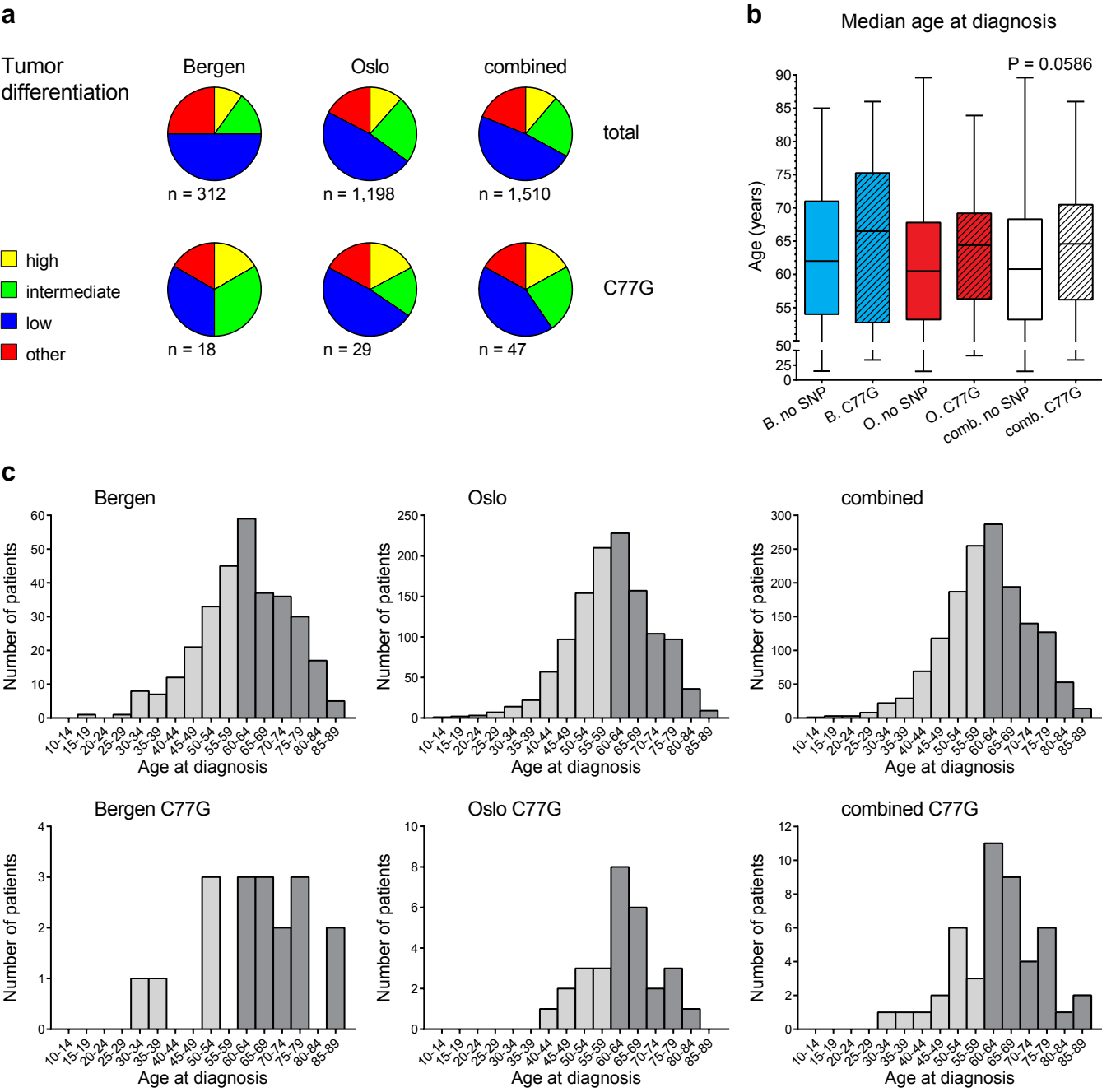

Supplement: S1 Fig — Distribution of tumour differentiation (a) and age at diagnosis (c) within the Bergen, Oslo and combined cohorts are displayed both for the respective total number of individuals (upper panels) and the C77G carriers (lower panels). Both, tumour differentiations and age at diagnosis, show similar distributions between the various cohorts. Light grey bars in (c): patients below the age 60, dark grey bars in (c): patient with an age above 60 years. (b) Median age at diagnosis of the Bergen (B., blue boxplots), Oslo (O., red boxplots) and combined (comb., white boxplots) cohorts is approx. four years higher in C77G-positive (striped plots) compared to C77G-negative patients. Boxes range from the first to the third quartiles. The medians are indicated by horizontal lines. Age minimums and maximums are displayed as whiskers. Combined cohorts “no SNP” vs. “C77G”: P = 0.0586. (PDF) [file pone.0182030.s001.pdf]

Supplementary Figure S2

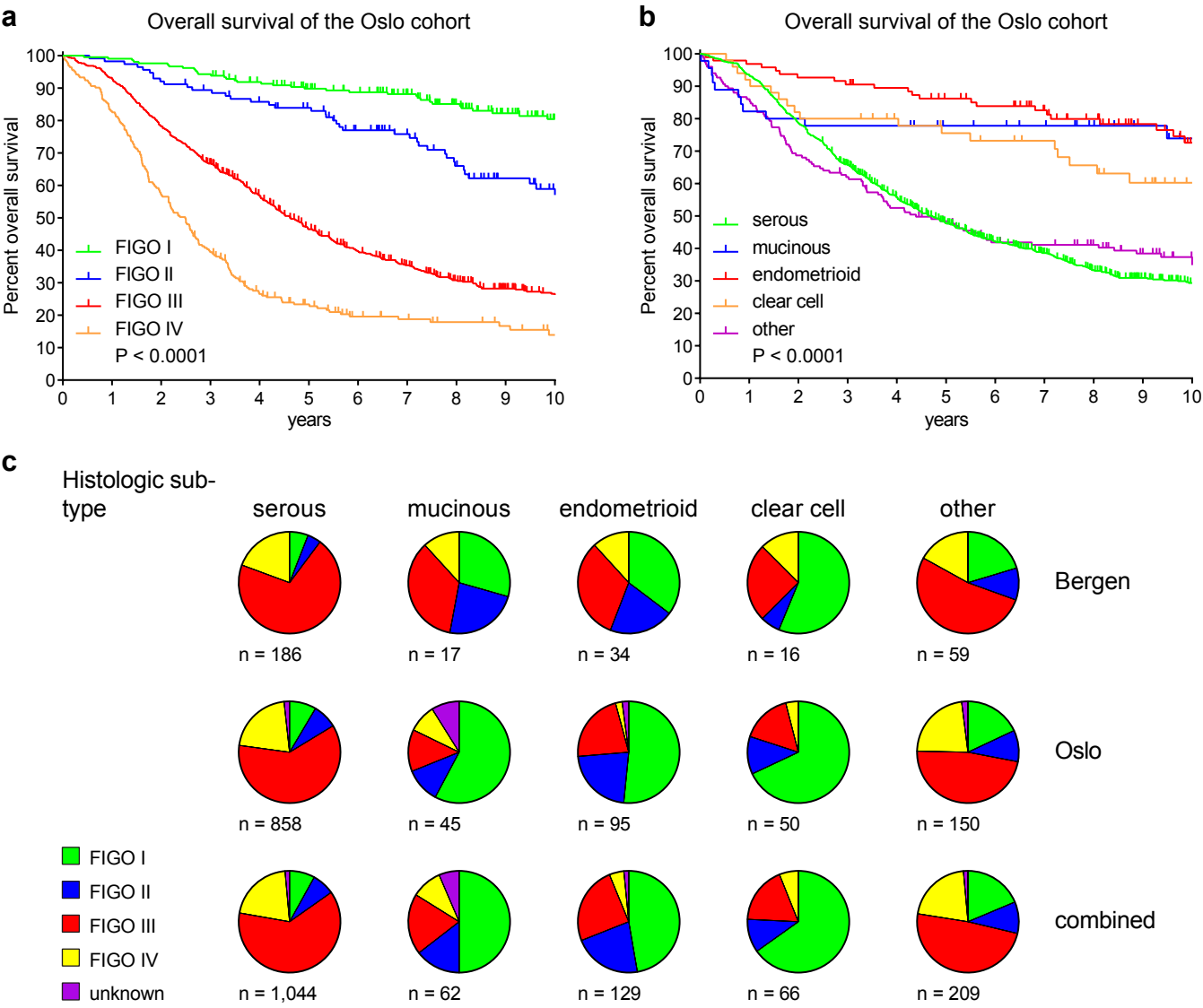

Supplement: S2 Fig — Overall survival curves for the different FIGO stages (a) and histological subgroups (b) within the Oslo cohort. Patients with FIGO stages I and II, and endometrioid or clear cell histologic subtypes have a relatively favourable prognosis. (c) Distribution of the FIGO stages within patient groups having a serous, mucinous, endometrioid, clear cell or different (“other”) histologic subtype for the Bergen, the Oslo and the combined cohorts. (PDF) [file pone.0182030.s002.pdf]
