# Supplementary material for: C77G in PTPRC (CD45) is no risk allele for ovarian cancer, but associated with less aggressive disease
Source: PLoS One. 2017 Jul 31;12(7):e0182030. doi: 10.1371/journal.pone.0182030 (PMC5536273; doi:10.1371/journal.pone.0182030)
Supplement: S1 Table — (PDF) [file pone.0182030.s003.pdf]

S1 Table. Clinicopathologic parameters of the cohorts.

	Bergen			Oslo			combined cohorts		
**FIGO**	**no SNP**	**C77G**	**MAF**	**no SNP**	**C77G**	**MAF**	**no SNP**	**C77G**	**MAF**
**FIGO I**	96	2	0.0204	411	7	0.0167	507	9	0.0174
**FIGO II**	48	4	0.0769	222	6	0.0263	270	10	0.0357
**FIGO III**	355	11	0.0301	1,244	12	0.0096	1,599	23	0.0142
**FIGO IV**	106	2	0.0185	442	4	0.0090	548	6	0.0108
**unknown**	0	0	0.0000	48	0	0.0000	48	0	0.0000
	**Bergen**			**Oslo**			**combined cohorts**		
**histology**	**no SNP**	**C77G**	**MAF**	**no SNP**	**C77G**	**MAF**	**no SNP**	**C77G**	**MAF**
**serous**	364	8	0.0215	1,695	21	0.0122	2,059	29	0.0139
**mucinous**	32	2	0.0588	88	2	0.0222	120	4	0.0323
**endometrioid**	63	5	0.0735	186	4	0.0211	249	9	0.0349
**clear cell**	32	0	0.0000	99	1	0.0100	131	1	0.0076
**other**	114	4	0.0339	299	1	0.0033	413	5	0.0120
	**Bergen**			**Oslo**			**combined cohorts**		
**differentiation**	**no SNP**	**C77G**	**MAF**	**no SNP**	**C77G**	**MAF**	**no SNP**	**C77G**	**MAF**
**high**	59	3	0.0484	271	5	0.0181	330	8	0.0237
**intermediate**	88	6	0.0638	557	5	0.0089	645	11	0.0168
**low**	305	7	0.0224	1,128	14	0.0123	1,433	21	0.0144
**other**	153	3	0.0192	411	5	0.0120	564	8	0.0140
	**Bergen**			**Oslo**			**combined cohorts**		
**age**	**no SNP**	**C77G**	**MAF**	**no SNP**	**C77G**	**MAF**	**no SNP**	**C77G**	**MAF**
**< 60 years**	507	5	0.0098	1125	9	0.0079	1,632	14	0.0085
**≥ 60 years**	721	15	0.0204	1242	20	0.0158	1,963	35	0.0175
**median age**	60.8	64.6		60.5	64.4		62.0	66.5	

Displayed are the numbers of C (no SNP) and G alleles (C77G), and the minor allele frequencies (MAF) within the different cohorts for the clinicopathologic parameters FIGO stage, histological subtype, tumour differentiation and age at diagnosis. Cells including the data of the homozygous patient are highlighted in yellow in the Bergen columns.
